# Revalidation of the spider genus *Citharoceps* Chamberlin, 1924 (Araneae, Segestriidae)

**DOI:** 10.3897/zookeys.495.8950

**Published:** 2015-04-08

**Authors:** André Marsola Giroti, Antonio Domingos Brescovit

**Affiliations:** 1 Departamento de Zoologia, Instituto de Biociências, Universidade de São Paulo, Rua do Matão, travessa 14, 321, 05508-090, São Paulo, São Paulo, Brazil; 2 Laboratório Especial de Coleções Zoológicas, Instituto Butantan, Avenida Vital Brazil, 1500, 05503-090, São Paulo, São Paulo, Brazil

**Keywords:** Taxonomy, Haplogynae, Synspermiata, Dysderoidea, California, stridulatory apparatus

## Abstract

*Citharoceps* Chamberlin was diagnosed by the presence of a very distinctive stridulatory apparatus composed of two patches of ridges on the sides of the cephalic region, and a stridulatory thorn on the prolateral region of the femur I. Currently, this genus is a junior synonym of *Ariadna* Audouin, with the assumption that the stridulatory apparatus could constitute an exclusive feature of its unique known species, *Citharoceps fidicina* Chamberlin, currently senior synonym of *Citharoceps californica* Chamberlin & Ivie. In the present study, *Citharoceps* is revalidated and redescribed based on the occurrence of the stridulatory apparatus in *Citharoceps fidicina* and *Segestria cruzana* Chamberlin & Ivie, and also on the presence of distinguishable characters, such as the length of the labium-sternum junction, ventral median spine on male metatarsi I, and strong sclerotized interpulmonary fold in females, forming a conspicuous median flap. *Segestria cruzana* is transfered to *Citharoceps*, with *Citharoceps californica* removed from the synonym of *Citharoceps fidicina*, and proposed as a junior synonym of *Citharoceps cruzana*, due to the similarity between the additional material examined and the original description. Males of *Citharoceps fidicina* and *Citharoceps cruzana* are described for the first time.

## Introduction

Segestriidae currently comprises 120 species distributed in three genera: *Segestria* Latreille, 1804, *Ariadna* Audouin, 1826 and *Gippsicola* Hogg, 1900 (World Spider Catalog 2015). *Ariadna* is the most diverse genus, with 99 taxonomically valid species and four generic junior synonyms: *Pylarus* Hentz, 1842, synonymized by Emerton (1890), *Macedonia* Hogg, 1900, synonymized by Rainbow (1911), and *Segestriella* Purcell, 1904 and *Citharoceps* Chamberlin, 1924, both synonymized by Beatty (1970).

The genus *Citharoceps* was described by Chamberlin (1924) to include only *Citharoceps fidicina*, described based on an immature holotype from Ensenada, Baja California, Mexico. He distinguished this genus from other segestriids by the presence of a stridulatory apparatus composed of two distinctive patches of grooves on both sides of the cephalic region and a stridulatory thorn located on the prolateral region of femur I. Later, Chamberlin and Ivie (1935) described a new species, *Citharoceps californica*, based on immatures from Laguna Beach, California, USA, distinguishing it from *Citharoceps fidicina* by the larger size and darker coloration.

Beatty (1970) examined the holotype of *Citharoceps fidicina* and paratypes of *Citharoceps californica*, and synonymized *Citharoceps californica* with *Citharoceps fidicina*, and *Citharoceps* with *Ariadna*. After the study was submitted to publication, Beatty (1970: 485) discovered a male specimen of *Citharoceps fidicina* with stridulatory patches like those found on the female. To the author, this characteristic, together with other structural features, confirms his assumptions on the synonymy of *Citharoceps* with *Ariadna*. This comment was included in the publication as an addendum.

In the present study, the revalidation of the genus *Citharoceps* is proposed, based on the presence of the stridulatory apparatus, together with distinctive characters, such as the labium-sternum length equal or smaller than the endite-sternum junction, a ventral median spine in the metatarsi I of males, and the internal female genitalia with a strong sclerotized interpulmonary fold, forming a conspicuous median flap. The genus is redescribed with more detailed information on the morphological characters, mainly with regard to male and female genitalia. The male of *Citharoceps fidicina* is described for the first time. *Citharoceps californica* is removed from the synonym of *Citharoceps fidicina* and proposed as a junior synonym of *Segestria cruzana* Chamberlin & Ivie, 1935, together with the transfer of this species to *Citharoceps*, and the first description of its male.

## Materials and methods

The specimens examined are deposited in the American Museum of Natural History, New York (AMNH; L. Prendini), California Academy of Sciences, San Francisco (CAS; C. E. Griswold), Collection of the Cabrillo National Monument Park, San Diego (CNMP; K. Lombardo), Darrel Ubick collection, San Francisco (CDU), Instituto Butantan, São Paulo (IBSP; A. D. Brescovit), and Queensland Museum, Brisbane (QM; R. Raven). The morphological examinations and descriptions follow Grismado (2008) and were made under Leica MZ6 and MZ12 stereomicroscope. Spine notation was modified from Grismado (2008) with the absence of the term “apical” (ap), and description of the leg IV spination. Measurements are in millimeters. The male genitalia was divided into bulb and embolus by the narrowing of the spermatic duct, where it has a less sclerotized region (Figs 6A–B, 8A–C; see Lipke et al. 2014, fig. 10). The investigation of the internal female genitalia followed three steps: (I) dissection of the ventral anterior region of the abdomen; (II) digestion of the dissected material with Ultrazime® contact lenses cleaner enzyme in 1 tablet/5 ml distilled water for 24 hours; (III) posterior treatment with heated KOH for 20 minutes, according to Platnick et al. (1999). Spigot nomenclature followed Platnick et al. (1991) and Griswold et al. (2005). Illustrations were made under a Zeiss Axioscop 20, with a camera lucida attached. Photographs were taken with a Leica DFC 500 digital camera attached to a Leica MZ16A stereomicroscope. Extended focal range photos were composed with Leica Application Suite 3.3. For scanning electron microscopy (SEM) images, the body parts were dehydrated through a series of graded ethanol (80% to 100%), dried by critical-point drying method, mounted on metal stubs using adhesive copper tape and nail polish for fixation, and sputter coated with gold. SEM photographs were taken with a FEI Quanta 250 scanning electron microscope from the Laboratório de Biologia Celular of the Instituto Butantan, São Paulo. Abbreviations: AC–aciniform gland spigot; ALS–anterior lateral spinnerets; AR–anterior receptaculum; B–bulb; d–dorsal; DL–dorsal lobe; E–embolus; GD–glandular ducts plate; IF–interpumonary fold; LS–less sclerotized portion of the sperm duct; mAP–minor ampulate gland spigot; MAP–major ampulate gland spigot; p–prolateral; PI–piriform gland spigot; PLS–posterior lateral spinnerets; PMS–posterior median spinnerets; PR–posterior receptaculum; r–retrolateral; T–tracheal trunk; UE–uterus externus; v–ventral; VL–ventral lobe; vp–ventroprolateral; vr–ventroretrolateral. Geographical coordinates were obtained with Google Earth (Lat/Lon-WGS84).

Vouchers for comparative studies: *Segestria senoculata* (Linnaeus, 1758): DENMARK: *Zealand Island*: Tisvilde, Tisvildeleje (56°03'08"N; 12°05'05"W) (DMS), 1♂ 1♀ 3imm., 19-20.V.1991, C. Griswold & N. Scharff leg. (CAS 9032847). *Ariadna maxima* (Nicolet, 1849): CHILE: *Concepcion Province*: Estación Escuadrón [36°55'59"S; 73°09'00"W] (DMS), 2♂ 5♀ 3imm., 20.IX.1980, N. Cekalovic leg. (AMNH); *Santiago Province*: Santiago [33°26'16"S; 70°39'01"W] (DMS), 1♀, 20.X.2009, T. H. Kawamoto leg. (IBSP 166664). *Gippsicola* sp.: AUSTRALIA: *Queensland*: Massey Range, 4km W of Centre Bellender Ker, 1250m (17°16'S; 145°49'E) (DMS), 2♂, 9–11.X.1991, Monteith, Janetzi & Cook leg. (QM S91041); Bellender ker Range, Summit TV Station, 1560m [17°14'S; 145°52'E] (DMS), 1♀, 1–7.XI.1981, Earthwatch/Queensland Museum leg. (QM S30617).

## Taxonomy

### Segestriidae Simon, 1893

#### Citharoceps

Taxon classificationAnimaliaAraneaeSegestriidae

Chamberlin, 1924 gen. reval.

Citharoceps Chamberlin, 1924: 607.

##### Type species.

*Citharoceps fidicina* Chamberlin, 1924

##### Diagnosis.

**Figure 1.**
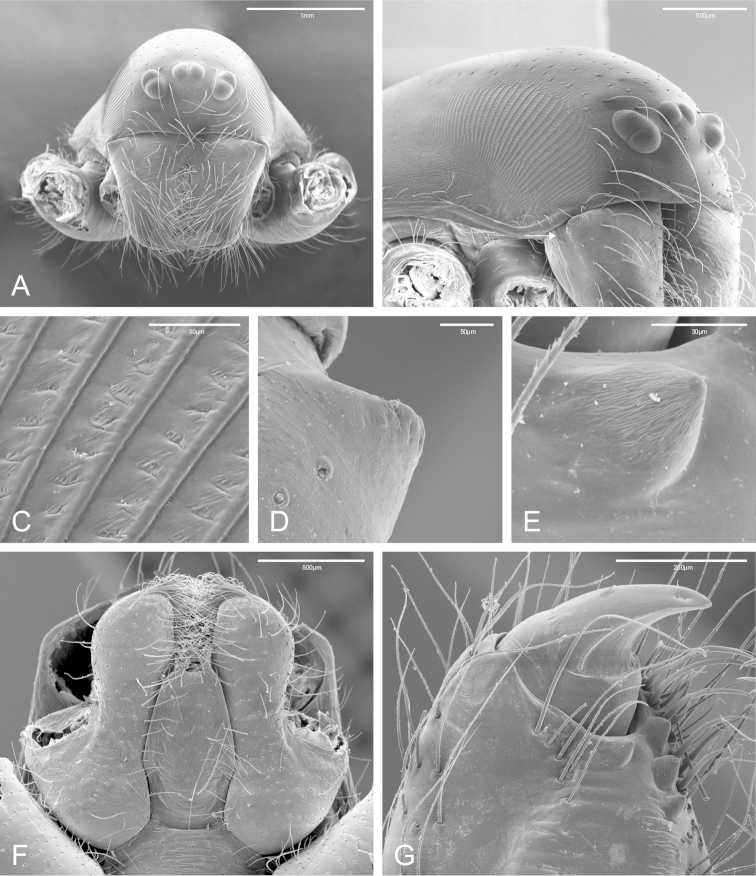
SEM images of *Citharoceps fidicina*, female from La Jolla, San Diego (CAS 9039517). **A** cephalothorax, frontal view **B** cephalothorax, frontal-lateral view, showing the right stridulatory patch, white arrow indicates the lateral proximal depression **C** stridulatory patch cuticle in detail **D** cheliceral lateral basal transverse ridge **E** cheliceral retromarginal tooth **F** endites and labium, ventral view **G** distal region of the right chelicerae, ventro-lateral view, showing the fang and the cheliceral teeth.

**Figure 2.**
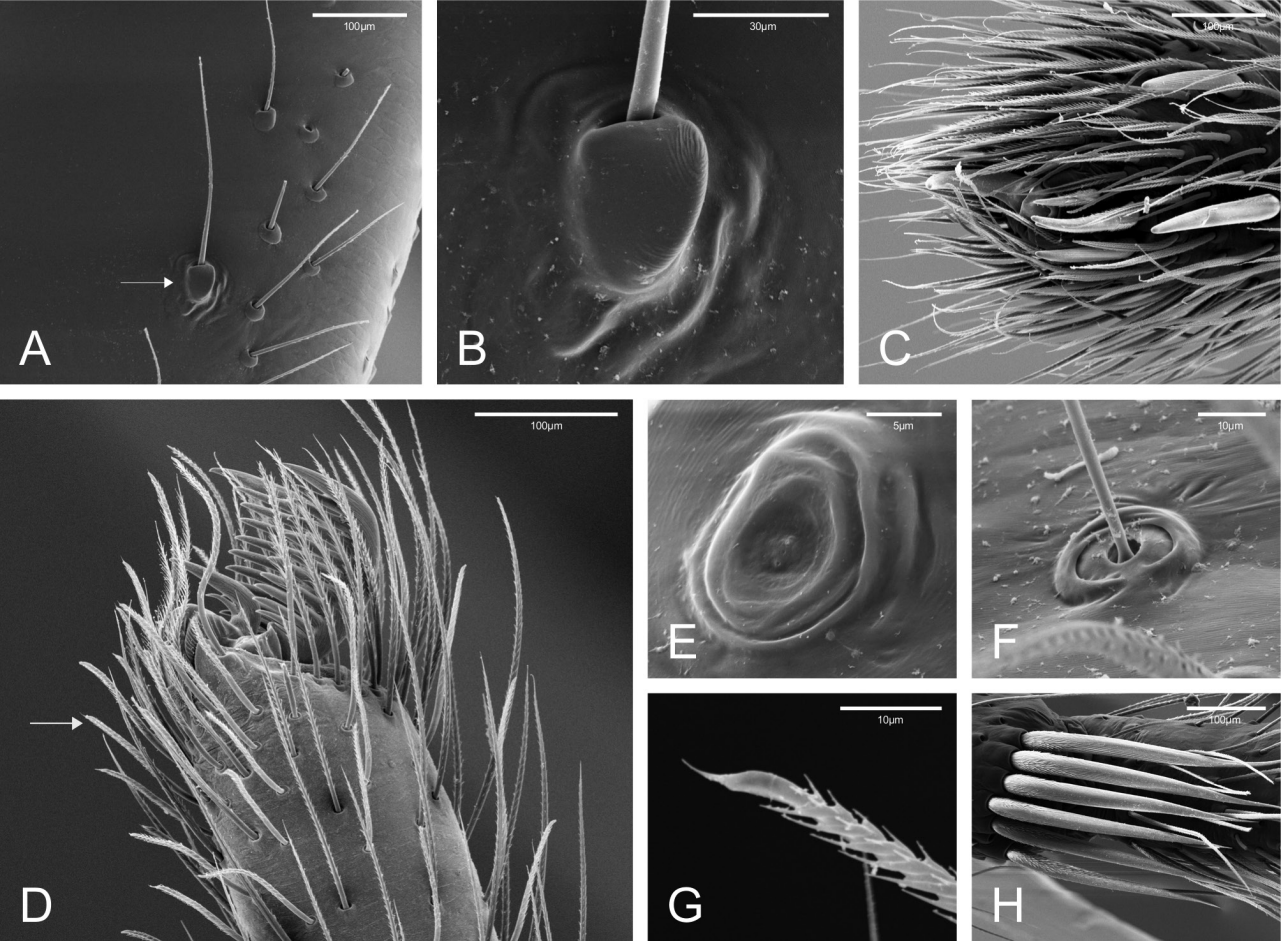
SEM images of *Citharoceps fidicina*, female from La Jolla, San Diego (CAS 9039517) (**A–C, E–F, H**) male from Cabrillo N. M., San Diego (CNMP) (**D, G**). **A** right femur I, median region, prolateral view, white arrow indicates the stridulatory thorn **B** stridulatory thorn, detail **C** right palp, ventro-prolateral view **D** left tarsus I, retrolateral view, white arrow indicates chemosensory setae **E** right tarsus II, tarsal organ **F** right tibia II, trichobothrial base **G** tarsal ventral chemosensory setae, detail **H** right metatarsus IV, distal preening comb, detail.

The genus *Citharoceps* is distinguished from other segestriid genera by the presence in males, females and immatures of a conspicuous stridulatory apparatus composed of two patches of grooves on both sides of cephalic region (Figs 1A–C) and a stridulatory thorn located on the prolateral region of femur I (Figs 2A–B; 5C, H; 7C, H). *Citharoceps* has a labium with the distal region narrowed (Fig. 1F; 9C), differing from *Segestria* (Fig. 9D; Giroti and Brescovit 2011: fig. 8) and *Gippsicola* (Fig. 9B), which have a nearly parallel-sided labium. It is distinguished from *Ariadna* (Fig. 9A) by the presence of a labium-sternum junction with equal or smaller length than the endite-sternum junction (Fig. 9C); by a ventral spine on the median region of male metatarsi I (Figs 5E; 7E), and by the females with an interpulmonary fold strongly sclerotized, forming a conspicuous median flap (Figs 4D–E; 6C; 8D), which is absent in *Ariadna* (Fig. 10A).

##### Description.

**Figure 3.**
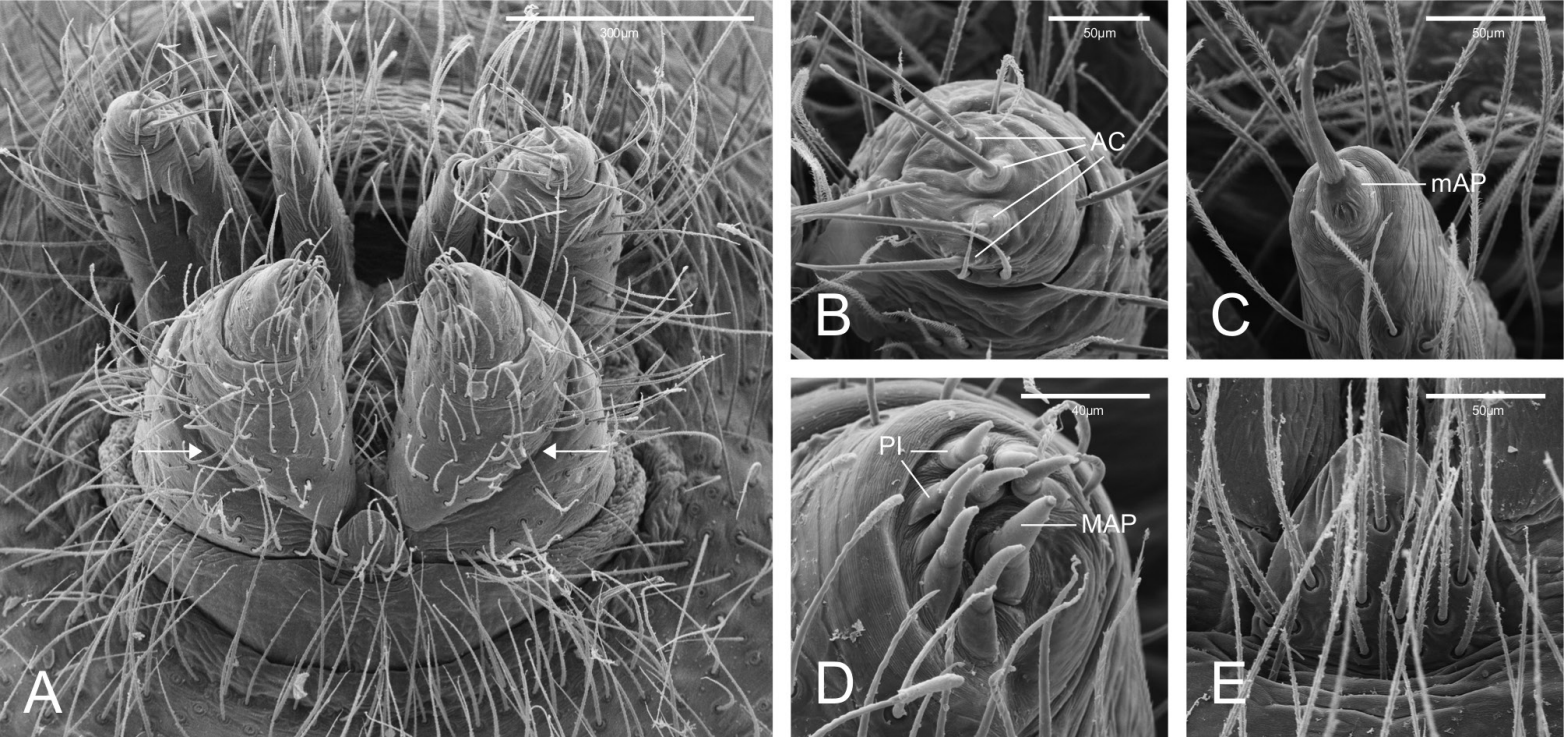
SEM images of *Citharoceps fidicina*, female from La Jolla, San Diego (CAS 9039517). **A** Spinnerets, ventro-posterior view, white arrows indicate the ALS basal segment transverse membrane **B** right PLS, posterior view **C** left PMS, posterior view **D** left ALS, posterior view **E** colulus in ventral view.

**Figure 4.**
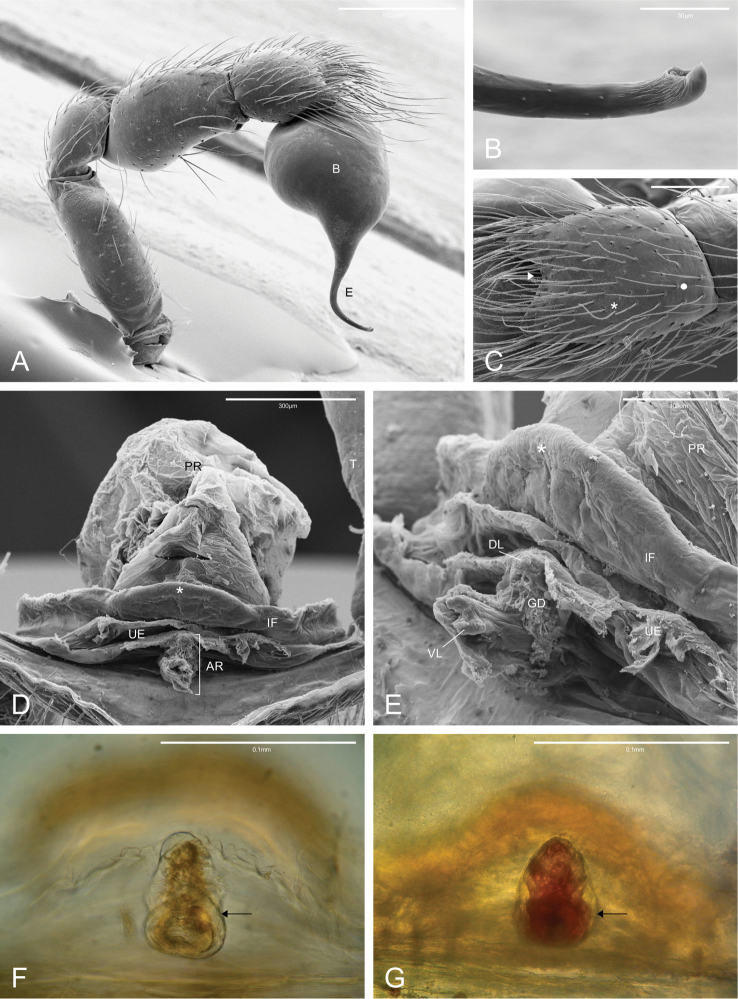
Male and female genitalia of *Citharoceps* species. *Citharoceps fidicina*, male from Cabrillo N. M., San Diego (CNMP) (**A–C**) female from La Jolla, San Diego (CAS 9039517) (**D–E**) and from Baja California, Mexico (CAS 9039518) (**F**) *Citharoceps cruzana*, female from Arroyo Seco, California (CDU) (**G**). **A** right palp, retrolateral view **B** embolus tip, prolateral view **C** cymbium, dorsal view, white arrow indicates distal notch, asterisk indicates a chemosensory setae and circle indicates a tactile setae **D** internal genitalia, apical view, and **E** lateral view, white asterisks indicates the median flap **F–G** anterior receptaculum, ventral view, black arrows indicate the hyaline cuticle.

**Figure 5.**
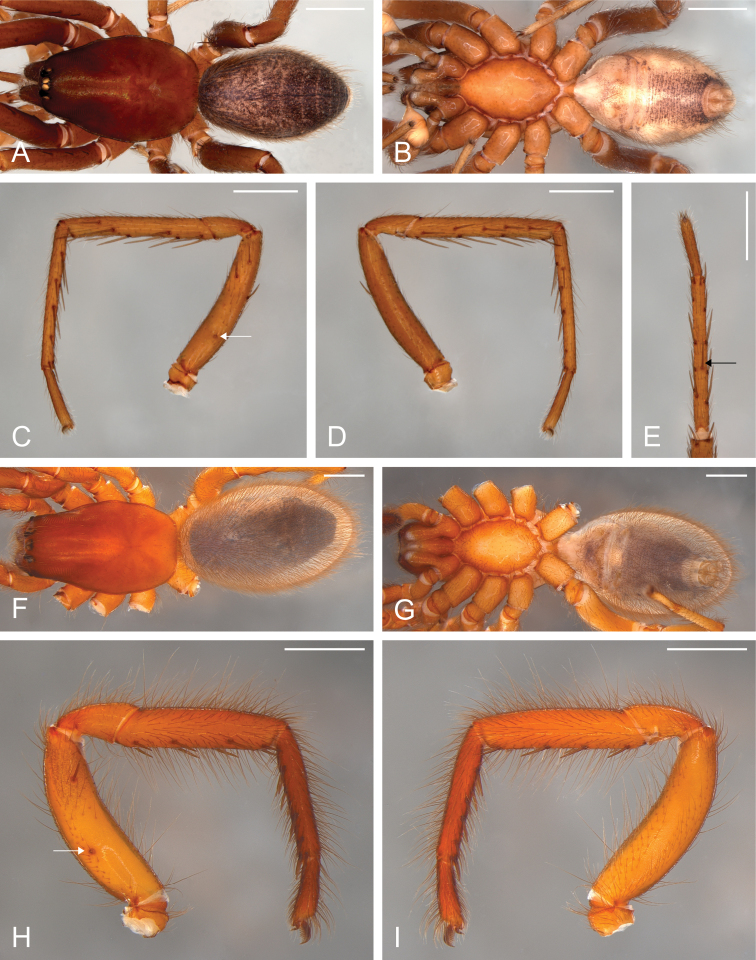
*Citharoceps fidicina*. Male from Cabrillo N. M., San Diego (CNMP) (**A–E**) female from La Jolla, San Diego (CAS 9039517) (**F–I**). **A, F** habitus, dorsal view **B, G** habitus, ventral view **C** right leg I, prolateral view **D** same, retrolateral view **E** right metatarsus and tarsus I, ventral view, black arrow indicates the ventral median spine **H** left leg I, prolateral view **I** same, retrolateral view; white arrows indicate the stridulatory thorn. Scale bars: 1 mm.

**Figure 6.**
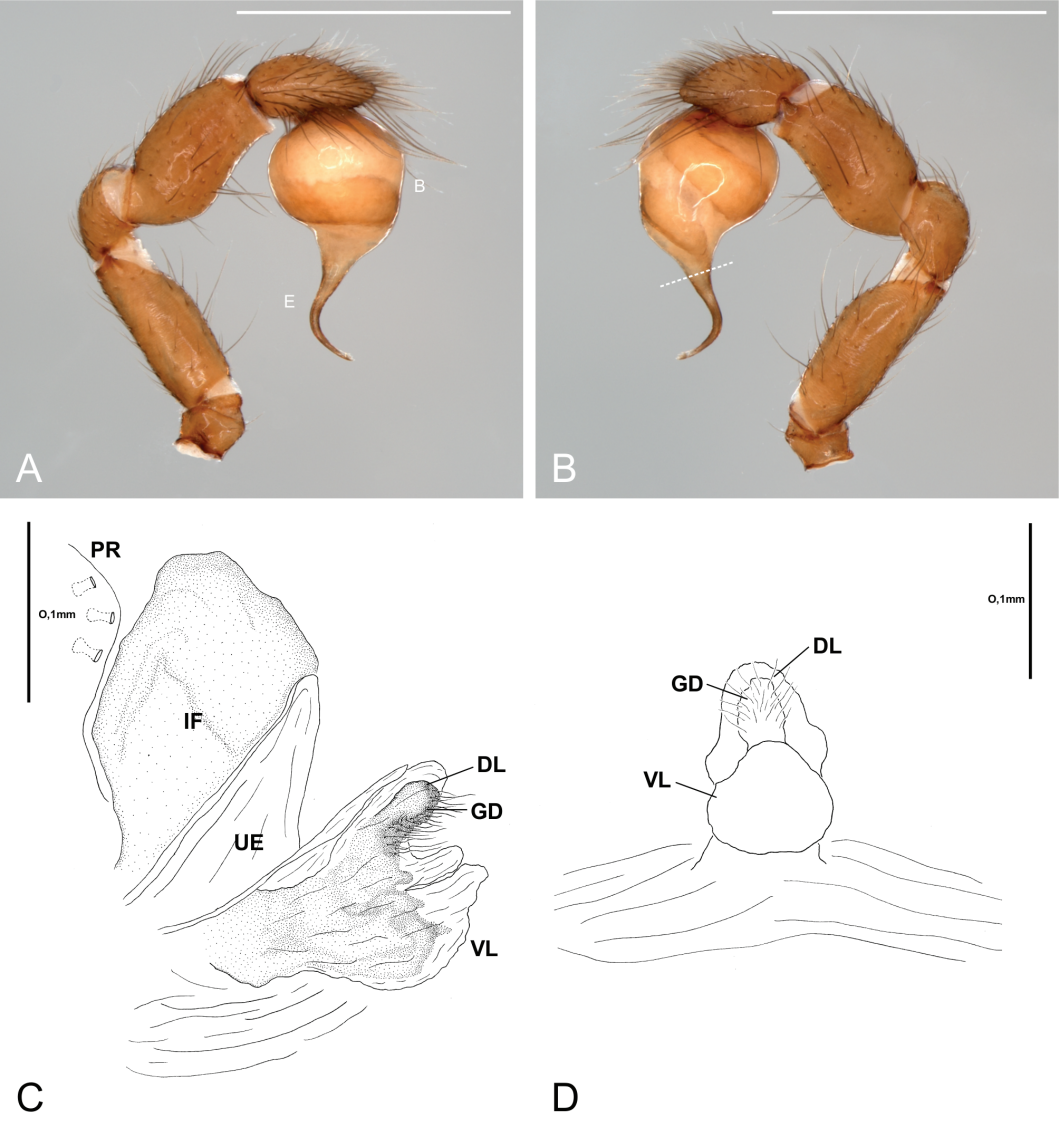
*Citharoceps fidicina*. Male from Cabrillo N. M., San Diego (CNMP) (**A–B**) female from Baja California, Mexico (CAS 9039518) (**C–D**). **A** left palp, prolateral view **B** same, retrolateral view, white dashed line indicates the narrowing of the sperm duct **C** internal female genitalia, lateral view, and **D** ventral view. White scale bars: 1 mm.

**Figure 7.**
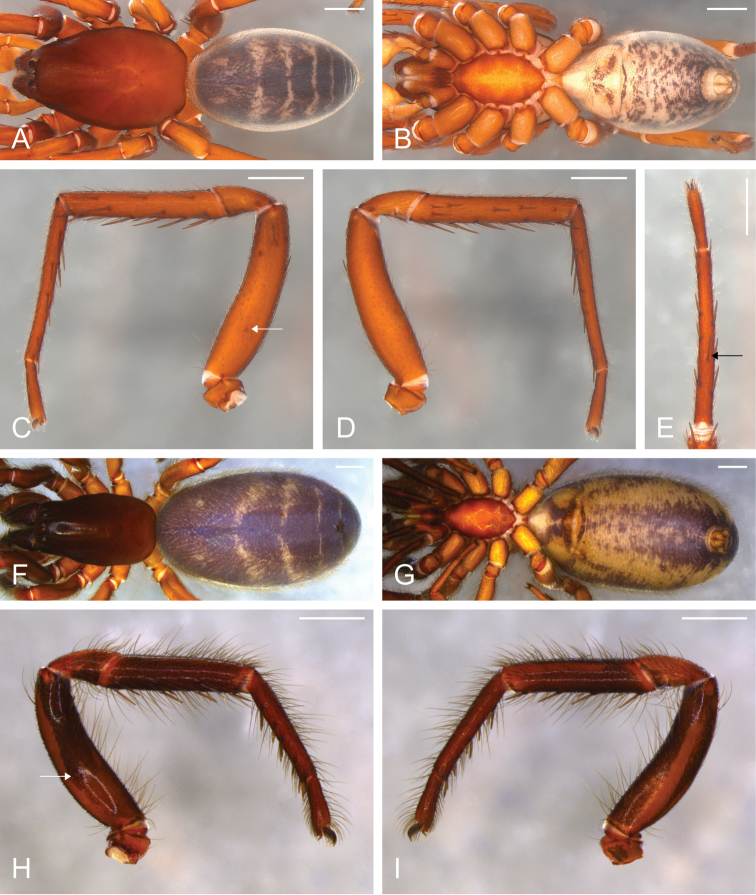
*Citharoceps cruzana*. Male from Santa Cruz Island, California (CAS 9046542) (**A–E**) female from Coast Ridge Trail, California (CDU) (**F–I**). **A, F** habitus, dorsal view **B, G** habitus, ventral view **C** right leg I, prolateral view **D** same, retrolateral view **E** right metatarsus and tarsus I, ventral view, black arrow indicates the ventral median spine **H** left leg I, prolateral view **I** same, retrolateral view; white arrows indicate the stridulatory thorn. Scale bars: 1 mm.

**Figure 8.**
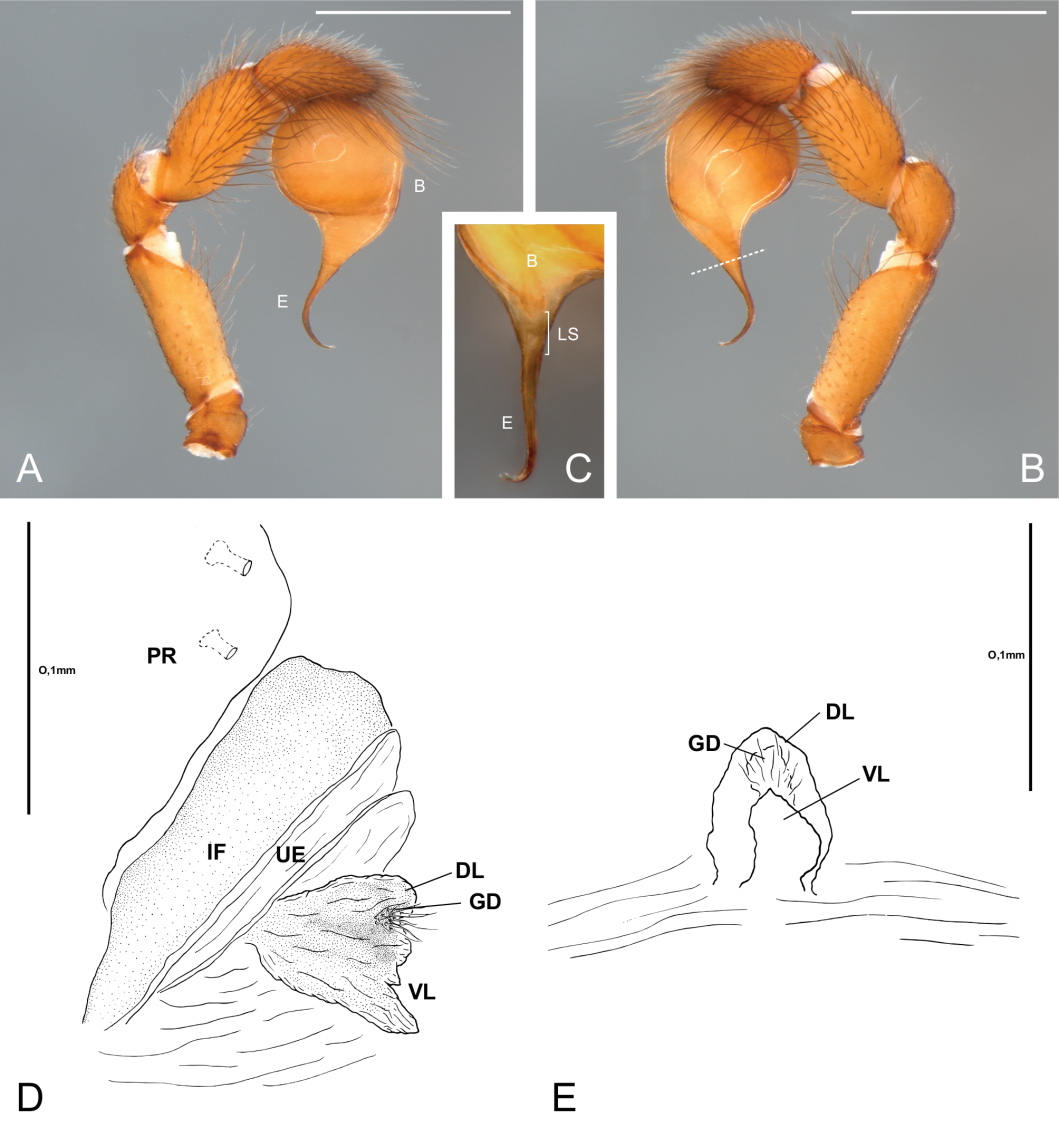
*Citharoceps cruzana*. Male from Santa Cruz Island, California (CAS 9046542) (**A–B**) male from Indians Road, Arroyo Seco Camping, California (CDU) (**C**) female from Arroyo Seco Camping, California (CDU) (**D–E**). **A** left palp, prolateral view **B** same, retrolateral view, white dashed line indicates the narrowing of the sperm duct **C** left palp, postero-retrolateral view, detail indicating the less sclerotized portion of the sperm duct **D** internal female genitalia, lateral view, and **E** ventral view. White scale bars: 1 mm.

**Figure 9.**
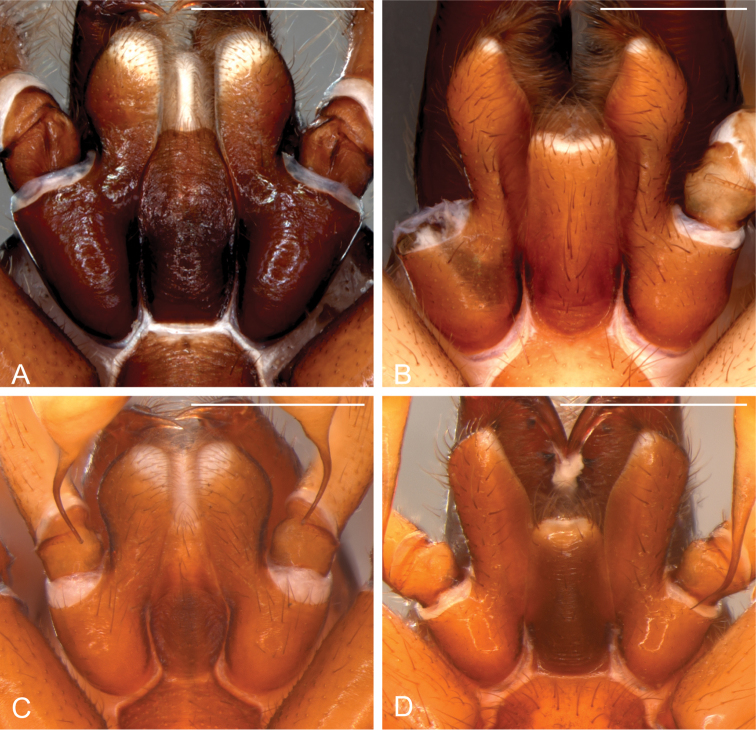
Endites and labium, ventral view. *Ariadna maxima*, male from Escuadrón, Concepción, Chile (AMNH) (**A**); *Gippsicola* sp., male from Massey Range, Queensland, Australia (QM S91041) (**B**); *Citharoceps cruzana*, male from Santa Cruz Island, California (CAS 9046542) (**C**); *Segestria senoculata*, male from Tisvildeleje, Zealand Island, Denmark (CAS 9032847) (**D**). Scale bars: 1 mm.

Medium-sized synspermiata spiders (see Michalik and Ramírez 2014). Total length 5.0–11.92, carapace 2.64–4.44 long, 1.76–2.76 wide. Carapace and chelicerae coloration ranging from orange to dark reddish orange. Eyes bounded by dark outlines (Figs 5A, F; 7A, F). Endites orange to reddish orange; labium dark orange to dark reddish orange. Sternum orange to reddish orange, with darker margins (Figs 5B, G; 7B, G). Legs orange to reddish orange with pair I–II darker. Femur, patella and tibia I–II distally marbled with darker pigment (Figs 5C–E, H–I; 7C–E, H–I). Abdomen greyish, with a dorsal pattern composed by dark chevrons, and irregularly distributed dark spots on the ventral region (Figs 5A–B, F–G; 7A–B, F–G). Carapace oblong, with cephalic region narrower than thoracic region, and sparsely distributed setae usually concentrated in the cephalic region. Posterior eyes positioned in a slightly recurved line (Figs 1A–B; 5A, F; 7A, F). Chelicerae: with prominent basal lateral ridge (Fig. 1D), and lateral proximal depression near the carapace (Fig. 1B); cheliceral teeth composed by three promarginal and one retromarginal, all with ridged cuticle (Figs 1E, G). Labium with 2/3 of the length of the endite, separated from the sternum by a partially membranous suture (Figs 1F, 9C; Labarque and Ramírez 2012: 6). Sternum longer than wide, with anterior region truncated and procurved anterior margin (Figs 5B, G; 7B, G). Male palp with a short and distally notched cymbium, piriform bulb and a hook-like embolus, with small tubercles (Fig. 4A–C). Female palps with one articulated claw, followed by scattered prolateral spines (Fig. 2C). Legs robust (Figs 5C–D, H–I; 7C–D, H–I). Tarsal organ exposed (following Labarque and Ramírez 2012) with edges, and usually three rimmed receptors (Fig. 2E; following Platnick et al. 2012). Trichobothria on the dorsal subdistal region of metatarsi I–IV, dorsal subproximal and subdistal region of tibia I–IV, and dorsal region of the male and female palpal tibia; trichobothrial bases with a transverse ridge, curved inwards; trichobothrial shaft filiform (Fig. 2F). Legs with three tarsal claws, paired claws pectinated, and unpaired one with only a small tooth (Fig. 2D); chemosensory setae on the distal ventral region of tarsi I–II in males (Fig. 2D, G; according to Foelix and Chu-Wang 1973: figs 17a–b). Preening comb of metatarsi IV with 5-7 spines (Fig. 2H). Abdomen uniformly hairy, longer than wider (Figs 5A–B, F–G; 7A–B, F–G). Spinnerets: ALS with three segments, the basal segment crossed by a diagonal membranous stripe (Fig. 3A), and one MAP spigot with 8 PI (Fig. 3D); PMS with just one mAP spigot (Fig. 3C); PLS with 4 AC spigots (Fig. 3B). Colulus triangular and pilose (Fig. 3E). Internal female genitalia: anterior receptaculum bilobated with a hyaline external cuticle, a short dorsal lobe, and a small plate of glandular ducts restricted to the ventral and lateral region of the dorsal lobe; posterior receptaculum membranous, with pores (Figs 4D–G; 6C–D; 8C–D).

#### Citharoceps fidicina

Taxon classificationAnimaliaAraneaeSegestriidae

Chamberlin, 1924 comb. rest.

Figs 1 , 2 , 3 , 4A–F , 5 , 6

Citharoceps fidicina Chamberlin, 1924: 608 (Immature holotype from Ensenada [31°51'28"N; 116°36'21"W] (DMS), Baja California, Mexico, 4.VII.1921, Soiver leg., deposited in CAS 1392, examined).
Ariadna fidicina – Beatty, 1970: 478 (Syn.). – World Spider Catalog, 2015.

##### Additional material examined.

**UNITED STATES OF AMERICA. *California*:** *San Diego County*: San Diego, La Jolla [32°50'24"N; 117°16'37"W] (DMS), Eucalyptus grove, 1♀ 1imm., XII.1965, F. Ennik leg. (CAS 9039517); San Diego, Point Loma Peninsula [32°41'1.64"N; 117°14'52.07"W] (DMS), 2♂, 8-12.IX.2003, D. Chan & T. Duffield leg. (CNMP); **MEXICO. *Baja California del Norte*:** 2.5mi S. of the Halfway House on Mex. Highway #1 (RB-3) [30°33'N; 115°10'W] (DMS), 2♀ 1imm., 21.XI.1962, P. R. Craig & D. Dailey leg. (CAS 9039518; 9039526; 9039546).

##### Diagnosis.

Males of *Citharoceps fidicina* differ from those of *Citharoceps cruzana* by the slightly shorter and thicker embolus (Fig. 6A–B) and by a straight metatarsus I (Fig. 5C–E). Females differ from *Citharoceps cruzana* by the anterior receptaculum with both dorsal and ventral lobes well delimited, the ventral lobe projected and laterally expanded (Figs 4D–F, 6C–D).

##### Description.

**Male (CNMP).** Total length 5.2; carapace 2.64 long, 1.76 wide. Palps light brownish orange (Fig. 6A–B). Legs: robust, with short setae, and spines with different sizes (Fig. 5C–E). Leg formula: I/II-III-IV. Leg measurements: I, femur 2.36, patella 0.88, tibia 1.96, metatarsus 2.12, tarsus 0.88, total 8.2; II, 2.36, 0.88, 1.96, 2.12, 0.88, 8.2; III, 1.88, 0.68, 1.4, 1.48, 0.68, 6.12; IV, 2.16, 0.88, 1.68, 1.64, 0,68, 7.04. Spination: leg I, femur d0-0-0-1-0-0/1-0, p0-0-0-0-1/0-1-0; tibia p0-0-0-1-0-1-0, vp1-0-1-0-1-0-1, v1-0-1-0-1-0-0, vr2/1-0-1-0-1-0-1, r0-1-1-1-1-1-0; metatarsus p0, vp0-1-0-0-1-1-1, v0-0-1-0-0-0-2, vr0-1-0-0-1-1-1, r0-1-0-0-0-0-0 (Fig. 5C–E); leg II, femur d0-0-1-1-0-0/1-0, p0-0-0-0-0-1-0; tibia p0-1-0-1-0-1-0, vp0-0-0-0/1-0-1-1, v0-0-1-0-1-0-0, vr1-0-1-0-1-0-1, r1-0-0/1-0-1-0-0; metatarsus vp0-1-0-0-1-1-1, v0-0-1-0-0-0-2, vr0-1-1-1-0-0-1; leg IV femur d1-0-1-0-1-0-0-0; tibia v0-0-0/1-0-0-1-0, vr0-0-0-0-0-0-1; metatarsus vp0-0-1-0-0-0-1, vr metatarsal comb with 5 spines.

**Female (CAS 9039517).** Total length 8.0; carapace 3.72 long, 2.36 wide. Palps orange, gradually darkening distally. Legs: robust, with long setae, and with ventral tibial and metatarsal spines of similar size (Fig. 5H–I). Leg formula: I-II-IV-III. Leg measurements: I, femur 2.56, patella 1.08, tibia 1.88, metatarsus 1.72, tarsus 0.76, total 8.0; II, 2.36, 1.08, 1.84, 1.76, 0.76, 7.8; III, 1.92, 1.0, 1.28, 1.24, 0.68, 6.12; IV, 2.4, 1.24, 1.8, 1.52, 0.68, 7.64. Spination: I, femur p0-0-0-0-0-1-0; tibia vp1-0-1-0-1-0-1, vr1-0-1-0-1-0-1; metatarsus vp0-1-0-1-1-1-1-1, v0-0-1-0-0-0-0-2, vr0-1-0-1-1-0-1-1-1 (Fig. 5H–I); II, tibia vr1-0-1-0-1-0-2/1; metatarsus vp0-1-0-1-1-1, v0-0-0/1-0-0-2, vr0-1-0-1-0-1; IV, vr metatarsal comb with 6-7 spines (Fig. 2H).

##### Variation.

Male (n=2): Total length 5.0–5.2; carapace 2.64–2.72 long; femur I 2.36–2.44. Female (n=3): Total length 6.08–8.0; carapace 3.0–3.72 long, 1.84–2.36 wide; femur I 1.92–2.56.

##### Distribution.

South of California, USA and Baja California, Mexico (Fig. 11).

##### Remarks.

We feel that species attribution is unproblematic. The immature specimen collected in Baja California del Norte by Craig and Dailey in 1962, matches perfectly with the type material of *Citharoceps fidicina*. In addition, that immature was collected with two females that match perfectly with the females collected in San Diego.

#### Citharoceps cruzana

Taxon classificationAnimaliaAraneaeSegestriidae

(Chamberlin & Ivie, 1935) comb. n.

Figs 4G , 7 , 8 , 9C

Segestria cruzana Chamberlin & Ivie, 1935: 7 (Immature holotype from Santa Cruz Island (34°00'N; 119°45'W) (DMS), Santa Barbara County, California, USA, III–IV.1913, R. V. Chamberlin leg., deposited in AMNH, examined). – World Spider Catalog 2015.
Citharoceps californica Chamberlin & Ivie, 1935: 8, pl. 5, figs 32–33 (Immature holotype and four female paratypes from Laguna Beach (33°31'N; 117°46'W) (DMS), California, USA, following Beatty (1970: 478), should be deposited in AMNH, not located, according to E. Sorkin, in letter). – Beatty 1970: 478 – NEW SYNONYMY.

##### Additional material examined.

**UNITED STATES OF AMERICA. *California*:** *Monterey County*: Greenfield, Arroyo Seco Camping, talus area at The Lakes (36°14'N; 121°29'W) (DMS), 1♀, 22.II.2002, D. & S. Ubick leg. (CDU); Greenfield, Arroyo Seco Camping, Indians Road, el. 1126’ (36°13.9'N; 121°29.5'W) (DMS), 1♂ 2imm., 18.VI – 24.X.2004, D. & S. Ubick leg. (CDU); Coast Ridge Trail [36°08'26.32"N; 121°33'10.03"W] (DMS), 0.8 mi SE Nacimiento Rd., el. 3000’, 1♀ 2imm., 1.VI.1991, D. Ubick leg. (CDU); *San Luis Obispo County*: Cayucos [35°26'18"N; 120°53'37"W] (DMS), 300’, 1♀, 15.XI.1937, O. Bryant leg. (CAS 9039527); *Santa Barbara County*: gully SW of U. C. Field Station, 80-90m, Santa Cruz Island [33°59'59"N; 119°45'42"W] (DMS), 1♂ 1imm., 22.IV.1994, D. H. Kavanaugh leg. (CAS 9055018; 9046542).

##### Diagnosis.

Males of *Citharoceps cruzana* differ from those of *Citharoceps fidicina* by the presence of a relatively longer and slender embolus (Fig. 8A–B), and a slightly prolaterally bent metatarsus I (Fig. 7C–E). Females differ from *Citharoceps fidicina* by the darker coloration, and anterior receptaculum with both dorsal and ventral lobes with similar length, the ventral lobe not expanded (Figs 4G, 8C–D).

##### Description.

**Male (CAS 9046542).** Total length 8.4; carapace 4.0 long, 2.76 wide. Palps light orange (Fig. 8A–B). Legs: robust, with short setae, and spines with different sizes (Fig. 7C–E). Leg formula: II-I-III-IV. Leg measurements: I, femur 3.2, patella 1.28, tibia 2.68, metatarsus 3.04, tarsus 1.04, total 11.24; II, 3.2, 1.24, 2.96, 3.12, 1.04, 11.56; III, 2.72, 1.16, 2.12, 2.36, 0.88, 9.24; IV, 3.16, 1.32, 2.6, 2.6, 0,88, 10.56. Spination: I, femur d0-0-0-0-1-0/1-2-0, p0-0-0-0-0-2-0-0; tibia p0/1-1-0-1-0-1-0, vp1-0-1-0-1-0-1, v1-1-0-1-0-0-0, vr1-1-0-1-0-1-0-1, r2-1-0-1-1-0-1-0; metatarsus vp0-1-1-1-0/1-1-1, v0-0-1-0-0-0-0, vr0-1-0-1-1-0-1-2, r0-1-0-0-0-0-0 (Fig. 7C–E); II, femur d1-0-1-0-1-1-2-0, p0-0-0-0-0-0-1-0; tibia p0/1-1-0-1-0-1-0, vp0-1-0-1-0-1-0-1, v1/0-0-1-0-0-1-0-0, vr1-1-0-1-1-1-1, r1-1-0-1-1/0-1-1; metatarsus vp0-1-1-1-0-1-1, v0-1/0-1-0-0-0-0, vr0-1-1-0-1-0-1/0-2, r0-1-0-0-0-0-0-0; IV, femur d1-1-0-1-0-1-0-1-0-0, r0-0-0-0-0-0-0-0-1-0; tibia vp0/1-1-0-0-1-0-0, vr0-0-0-0-0-1/0-1; metatarsus vp0-0/1-1-0-0-1-0-1, metatarsal comb with 6 spines.

**Female (Coast Ridge Trail, CDU).** Total length: 11.92; carapace 4.44 long, 2.6 wide. Palps reddish orange, gradually darkening distally. Legs reddish orange, with pairs I-II darker. Femur, patella and tibia I-II darkly marbled mainly on the ventral, prolateral and retrolateral regions (Fig. 7H–I). Legs: robust, with long setae, and with ventral tibial and metatarsal spines of similar size (Fig. 7H–I). Leg formula: I-II-IV-III. Leg measurements: I, femur 2.88, patella 1.28, tibia 2.12, metatarsus 2.12, tarsus 0.88, total 9.28; II, 2.84, 1.28, 2.16, 2.12, 0,84, 9.24; III, 2.32, 1.08, 1.44, 1.6, 0.76, 7.2; IV, 2.84, 1.28, 2.08, 1.8, 0.8, 8.8. Spination: leg I, femur p0-0-0-0-0-2-0; tibia vp1-0-1-0-1-0-1, vr1-0-1-0-1-0-1; metatarsus vp 0-1-1-1-1-1-1, v0-0-0-0-0-0-2, vr0-1-1-1-0-1-1 (Fig. 7H–I); leg II, femur p0-0-0-0-0-1-0; tibia vp0-0-0-0-0-0-1, vr1-0-1-0-1-0-1; metatarsus vp0-1-0-1-0-1-1, v0-0-0-0-0-0-2, vr0-1-0-1-0-1-1; leg IV, tibia vr0-0-0-0-0-0-1; metatarsus vp0-0-1/0-0-0-0-2/1, vr metatarsal comb with 5-6 spines.

##### Variation.

Male (n=2): Total length 8.0–8.4; carapace 3.92–4.0 long, 2.4–2.76 wide; femur I 3.08–3.2. Female (n=3): Total length 9.32–11.92; carapace 4.24–4.44 long, 2.6–2.76 wide; femur I 2.72–2.92.

##### Distribution.

Monterey County to Santa Barbara County, California, USA (Fig. 11).

##### Remarks.

One male of *Citharoceps* was found from the type locality of *Segestria cruzana*. After the examination of the immature holotype of *Segestria cruzana*, it was detected that it possessed the stridulatory apparatus exclusive for *Citharoceps*, not detected by Chamberlin and Ivie (1935), thus we transferred this species to this genus. In addition, in the additional material, females of *Citharoceps* were found from the middle coast of California that resemble those described by Chamberlin and Ivie (1935) as *Citharoceps californica*, together with a male, from the same region, that is similar to the one from Santa Cruz Island. Thus, *Citharoceps californica* is removed from its synonym with *Citharoceps fidicina*, due to its greater size and darker coloration, and placed as junior synonym of *Citharoceps cruzana*.

## Discussion

**Figure 10.**
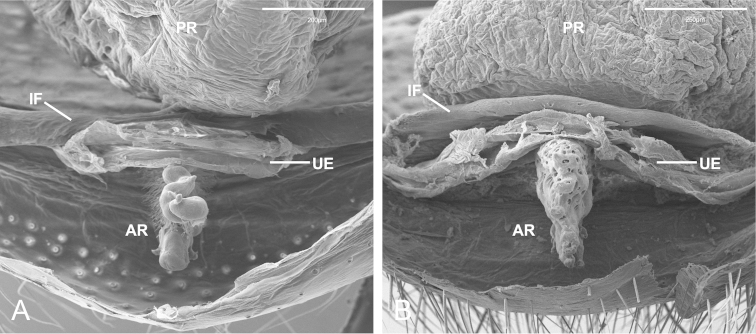
*Ariadna maxima*, female from Santiago, Chile (IBSP 166664) (**A**); *Gippsicola* sp., female from Bellender ker Range, Queensland, Australia (QM S30617) (**B**); SEM images of internal female genitalia, apical view, showing the absence of the median flap.

**Figure 11.**
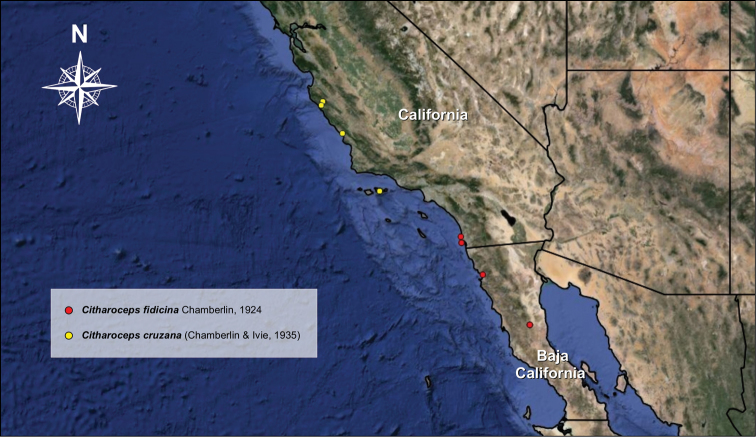
Locality of the specimens of *Citharoceps* examined in the present study.

The distribution of the genus *Citharoceps* Chamberlin, 1924 comprises only coastal regions of the state of California, USA, and Baja California, Mexico (Fig. 11). The Baja California peninsula has a tectonical origin and is well known to harbor a great diversity and endemic species of fauna and flora, mainly because of climate and topography divergences (Murphy 1983, Grismer 1994). Under this scenario, the diagnostic features presented by *Citharoceps* are rather distinct based on the other two segestriid genera occurring in this region e.g. *Ariadna* and *Segestria*. Although those characters are putative synapomorphies, the stridulatory apparatus, once thought by Beatty (1970) to be exclusive of *Citharoceps fidicina*, is also present in *Citharoceps cruzana*, contradicting his assumption. In addition, the labium-sternum junction length, the presence of a ventral median spine on metatarsi I of males, and the morphology of the interpulmonary fold are very distinctive characters from *Segestria* (Fig. 9D; Giroti and Brescovit 2011, figs 19–20, 23), *Ariadna* (Figs 9A, 10A; Grismado 2008, figs 1A, 4D, 6H) and *Gippsicola* (Figs 9B, 10B). Under these circumstances, it seems reasonable that *Citharoceps* is a different genus and must be treated as a valid taxon.

### Notes on the stridulatory apparatus of *Citharoceps*

The occurrence of a stridulatory apparatus in spiders has been reported in at least 25 spider families (Legendre 1963, Rovner 1980, Uetz and Stratton 1982, Starck 1985, Maddison 1987, Wunderlich 1995, Ramírez et al. 2001, Jocqué 2005), and also phylogenetically tested in Haplogynae (Labarque and Ramírez 2012) and Entelegynae (Griswold et al. 2005, Ramírez 2014). Legendre (1963) was the first to classify these apparatus (types a–g), and Starck (1985) provided a complete list of known stridulatory apparatus in spiders, also discussing their structures and evolutionary context. The stridulatory apparatus found in *Citharoceps* can be classified as belonging to the “type l”, with ridges or grooves (*pars stridens*) in the carapace; and thorns (*plectron*) in the femur I. This type was also described by Maddison (1987) for seven salticid genera.

The function of the stridulatory apparatus in *Citharoceps* is unknown, but considering that it occurs in males, females and immatures, it seems unlikely that it has a courtship function. Maddison (1987) reported a personal comment of M. J. Moody having heard a sub-adult male of *Citharoceps fidicina* making a loud buzzing sound while rubbing the carapace ridges. Considering this information, the function of the stridulatory apparatus in *Citharoceps* could involve defensive buzzing sounds, as described by Uetz and Stratton (1982) for *Micrathena gracilis* (Walckenaer, 1805), but future ethological studies are needed to corroborate this hypothesis.

## Supplementary Material

XML Treatment for Citharoceps

XML Treatment for Citharoceps fidicina

XML Treatment for Citharoceps cruzana
